# Parent involvement in child anthropometric measurement

**DOI:** 10.1186/s12875-023-02028-2

**Published:** 2023-04-05

**Authors:** Sarah Rae, Frank Ong, Cindy-Lee Dennis, Jill Hamilton, Eleanor Pullenayegum, Jonathon Maguire, Catherine Birken

**Affiliations:** 1 Child Health Evaluative Sciences, The Peter Gilgan Centre for Research and Learning, The Hospital for Sick Children, Toronto, ON Canada; 2 Faculty of Nursing, University of Toronto, Toronto, ON Canada; 3 The Hospital for Sick Children, Toronto, ON Canada; 4 Department of Nutritional Sciences, University of Toronto, Toronto, ON Canada; 5 Division of Paediatrics, St Michael’s Hospital, Toronto, ON Canada; 6 Division of Paediatric Medicine, The Hospital for Sick Children, Toronto, ON Canada; 7 Division of Endocrinology, The Hospital for Sick Children, Toronto, ON Canada

**Keywords:** Child Health, Early childhood obesity, Primary care, Clinical Research, Anthropometric measurement, Parent participation, Parent perspective, Qualitative research, Mixed methods

## Abstract

**Background:**

Young children are often accompanied by their parent/caregiver when attending primary healthcare visits, where clinical procedures such as anthropometric measurements are conducted. Parents are not typically involved in their child’s anthropometric measurement collection, and there are no recommendations for parental involvement during visits. The objective of this study was to describe parents’ experiences with being involved in their child’s anthropometric measurements.

**Methods:**

A 10-question survey comprised of scaled and open-ended questions was self-administered to participants after child anthropometric measurement collection including length/height, weight, head, arm, and waist circumference. Survey data were analyzed using a general inductive approach and thematic analysis. Surveys were collected in participating TARGet Kids! primary care practice sites in Toronto, Canada. Survey respondents included 30 parents of children < 2 years of age, and 30 parents of children 2–5 years of age.

**Results:**

76% of parents with children aged < 2 years and 93% of those with children aged 2–5 years rated their overall experience in being involved in their child’s anthropometric measurement as enjoyable or thoroughly enjoyable. Analysis of open-ended survey questions revealed five themes: [1] parent interest in child growth; [2] ease of anthropometric measurement; [3] extended clinic visit; [4] child discomfort; and [5] interest in participating in research.

**Conclusion:**

Parents reported a high degree of enjoyment in being involved in their child’s anthropometric measurements. Parent participation in anthropometric measurement may improve parental satisfaction with children’s primary healthcare. Future research may include assessing the reliability of measurements taken with the support of a parent/caregiver.

**Supplementary Information:**

The online version contains supplementary material available at 10.1186/s12875-023-02028-2.

## Background

Young children are often accompanied by their parent or caregiver (referred to as parent) when attending primary healthcare visits, where clinical procedures such as anthropometric measurements are obtained. Parents are not typically involved in these clinical primary healthcare visits. The involvement of a child’s parent in anthropometric measurement during the primary healthcare encounter may improve parental satisfaction with the encounter, provide a sense of security and comfort for the child, and assist the healthcare professional in obtaining anthropometric measurements. Despite the importance of the parental role in the healthcare visit, no current literature is available surrounding the parent’s overall experience of being involved in their child’s clinical measurements or procedures. A systematic review which examined parental presence in pediatric ‘painful procedures’ found mixed results [1]. One study showed that parental participation helped reduce the pain a child may experience during an invasive procedure [2]. Another study in the pediatric intensive care unit (PICU) identified emerging themes in parent perception of their role including being present and participating in child care, forming partnership of trust with healthcare professionals, and being informed of child’s progress and treatment plan [3]. Measurement of a child’s height and weight is a recommended part of primary healthcare practice for children [4]. Understanding parent’s experience in assisting with anthropometric measurements may help improve parental satisfaction with the clinical encounter, minimize child discomfort and assist the healthcare provider in the collection of reliable anthropometric measurements.

The objective of this study was to explore parent’s experience with being involved in their child’s anthropometric measurements during a primary healthcare encounter. It was hypothesized that parents would report: [1] a high degree of enjoyment in being involved in their child’s anthropometric measurements; [2] the desire to be involved in their child’santhropometric measurement collection during their healthcare visit; and [3] an increased sense of security and comfort experienced by their child during the anthropometric measurement due to their involvement.

## Methods

### Study design and participants

This study was a part of a larger mixed method study using quantitative methods to determine inter- rater reliability of child anthropometric measurements between two trained research assistants compared to a trained research assistant and parent dyad, and survey and qualitative methods to understand parent experiences of involvement in anthropometric measurement [6]. Parents (including mothers and fathers) were recruited through the TARGet Kids! (The Applied Research Group for Kids) practice-based research network [5] that consists of a collaboration of child health researchers and primary care physicians (pediatricians and family physicians) in Toronto, Ontario, Canada.

Children were ineligible to participate if they had a chronic health condition(s) or severe developmental delay(s) [5]. Families who are unable to communicate in English were excluded [5]. Additional study exclusions included children who had health conditions that may have affected normal anthropometric measurements (i.e., non-ambulatory, leg contractures, or wearing a cast). Each participating child was accompanied by at least one parent or caregiver.

This study was conducted according to the guidelines laid down in the Declaration of Helsinki and all procedures involving research study participants were approved by the Research Ethics Board (REB) approval from The Hospital for Sick Children.

### Anthropometric measurement collection

Each child had their length/height, weight, head, arm, and waist circumference measured four times each, twice by the two trained research assistants and twice by the trained research assistant and parent, using the World Health Organization’s (WHO) standardized training for anthropometric measurements in primary care practice [7]. These guidelines differ by age; to measure the weight of a child who is two years of age or older, the WHO recommends using a durable, electronic (digital reading) scale [7]. To measure the weight of a child who is younger than two years of age the child is weighed using an electronic (digital) scale in the recumbent position [7]. For measurement of height, if the child is two years of age or older they had their standing height measured using a stadiometer. If the child is younger than two years of age, their recumbent length was measured using a length board [7]. For children younger than two years of age who used a length board, the individual who assisted in the measurement collection held the child’s head in place, held the child’s legs straight on a length board, and lastly, held the child’s arms in place [7]. Head circumference was collected while the child was seated on the parent’s lap. The measurer made a loop with the tape measure which was positioned over the child’s eyebrows and leveled across the front of the head. The greatest circumference of the head was located by moving the tape measure across the back of the protuberance of the skull at the back of the child’s head. Measurement was taken after pulling the tape measure tight to compress the child’s hair and skin. The measurement reading was recorded to the last completed 1 mm [7]. Arm circumference was taken by first locating the upper arm mid-point. The upper arm mid-point was located by marking the child’s acromion process on the left shoulder, bending the elbow at a right angle, and marking the mid-point of the acromion process and tip of the elbow. The measuring tape was passed over the mid-point, placed flat on the child’s skin without compressing tissue, and the measurement was recorded to the last completed 1 mm [7]. For children under 12 months of age or younger, waist circumference was measured lying down. To measure waist circumference, the child’s hip bones were first located. The measuring tape was placed around the child’s waist, right above the hip bones and came around to the belly button. The tape measure was held firmly and did not sag, and the measurement was recorded to the nearest 0.1 cm [7]. For children 2 years of age or older and who can stand, waist circumference was measured standing up. The child stood with their feet together, placed arms at their side, and breathed out gently. The tape measure was placed right above the child’s hip bones and sat parallel to the floor while lying snug on the child’s skin but did not compress the skin. The measurement was recorded to the nearest 0.1 cm [7].

### Survey design

The Parent Involvement Survey, was a brief 10-question survey, created by our clinical research team including qualitative researchers, research assistant and study coordinators, a pediatrician, and a parent (Table 1). The survey consisted of a combination of Likert-scaled, Yes/No, and open-ended questions and was designed to take no longer than five minutes to complete. On the Likert scale, a value of “1” represents that the participant does not agree with the question or believes the question to be true, whereas a value of “5” represents that the participant agrees with the question or believes the question to be true.

**Table 1:** Parent Involvement Survey responses of parents of children ages < 2 (*n =* 30) years and 2–5 years (*n =* 30)

	Children < 2 years (n = 30)	Children 2–5 years (n = 30)
Question	% (*n*)	% (*n*)
**How would you rate your overall experience in being involved with your child’s growth measurement?**		
1 – Did not enjoy	0	0 (0)
2	7 (2)	0 (0)
3	17 (5)	7 (2)
4	43 (13)	33 (10)
5 – Thoroughly enjoyed	33 (10)	60 (18)
**How comfortable would you say you were in assisting in your child’s growth measurements?**		
1 – Not comfortable at all	0	0 (0)
2	3 (1)	0 (0)
3	3 (1)	0 (0)
4	27 (8)	23 (7)
5 – Extremely comfortable	67 (20)	77 (23)
**Did being involved in your child’s growth measurements create any stress for you?**		
1 – I felt no stress	57 (17)	83 (25)
2	20 (6)	10 (3)
3	20 (6)	7 (2)
4	3 (1)	0 (0)
5 – I felt a great deal of stress	0 (0)	0 (0)
**Did being involved in your child’s growth measurements create any stress for your child?**		
1 – My child did not experience any stress	27 (8)	67 (20)
2	36 (11)	10 (3)
3	23 (7)	7 (2)
4	7 (2)	13 (4)
5 – My child experienced a lot of stress	7 (2)	3 (1)
**Do you believe that your involvement in your child’s growth measurements was able to create a sense of security and comfort for your child?**		
1- My child was not comfortable at all	3 (1)	0 (0)
2	17 (5)	3 (1)
3	13 (4)	3 (1)
4	47 (14)	24 (7)
5 – My child was extremely comfortable	20 (6)	70 (21)
**Have you been involved in assisting with your child’s measurements in the past?**		
Yes	43 (13)	40 (12)
No	57 (17)	60 (18)
**Would you like to be involved in your child’s growth measurements at their clinic visit in the future?**		
Yes	83 (25)	80 (24)
No	0 (0)	7 (2)
Maybe	17 (5)	13 (4)
**If yes, would you like to have lesser, greater or the same amount of involvement?**		
Lesser	0 (0)	0 (0)
Greater	12 (3)	0 (0)
Same	88 (22)	100 (24)

Parents were asked using open-ended questions to comment on their overall experience, what they did or did not enjoy about their involvement, and about their past/future involvement in their child’s primary healthcare visits.

### Sample size

Purposeful sampling was used per child age group (< 2, and 2–5 years), a sampling technique widely employed in qualitative research for the selection of individuals or groups that are knowledgeable or have experienced in the phenomenon of interest [8]. Data saturation was reached when the data appeared to be redundant, and there were no new emerging concepts or themes discovered from the parent responses to the survey questions [9].

### Data analysis

The analysis of the survey data was conducted using quantitative and qualitative approaches. The quantitative data consisted of the participant responses to both the Likert-scaled and Yes/No survey questions. The question responses were totaled, and corresponding percentages were calculated. In the qualitative analysis, participants were asked to answer open-ended questions regarding their overall experience in being involved in their child’s anthropometric measurements, what they enjoyed or did not, and their interest in future involvement. The surveys were separated based on age group (< 2 years and 2–5 years) and de-identified (names and identifiable information removed). The textual data were reviewed and analyzed by the primary researcher. A thematic question analysis and general inductive approach were used to analyze and interpret the textual data [10]. The text from the parent responses were coded and placed into specific categories, and overall themes were drawn across the responses to the open-ended survey questions [10]. The primary researcher first familiarized herself with the data by reading all the responses to the open-ended questions and grouping together similar responses. The text was then coded, based on whether the response was positive, negative, or discussed past/future involvement. After the text was coded, categories were created based on the similarity of the coded words. For instance, words that indicated an overall positive experience, were grouped together for further analysis. Once the text was put into categories, they were further analyzed for theme identification. The identified themes were then described, labeled, and developed based on the primary study objective. Themes were then reviewed and refined with the assistance of a qualitative expert and secondary reviewer, and supportive theme quotes were selected.

## Results

### Baseline characteristics

Baseline characteristics for all participants included in the present study sample were displayed in Table 2.

**Table 2:** Characteristics for study participants by child age group

Variable	Children of Parents (< 2 years) N = 30		Children of Parents (2–5 years) N = 30	
	Mean (SD)		Mean (SD)	
**Child Age (months) mean (SD)**	7.19 (6.03)		41.39 (10.94)	
**Parental Age (years)**	33.6 (7.47)		33.9 (4.69)	
	**Category**	***N*** **(%)**	**Category**	***N*** **(%)**
**Child Sex**	Male	11 (37)	Male	14 (47)
	Female	19 (63)	Female	16 (53)
**Parental Ethnicity**	European	4 (13)	European	11 (37)
	Asian	2 (7)	Asian	6 (20)
	Latin American	0 (0)	Latin American	1 (3)
	Mixed Ethnicity	4 (13)	Mixed Ethnicity	3 (10)
	Unreported	20 (67)	Unreported	9 (30)
**Parental Educational Attainment (%)**	Some College/University or above	29 (0.96)	Some College/University or above	29 (0.96)
	High school degree or below	1 (0.03)	High school degree or below	1 (0.03)
**Self-Reported Family Income**	< $100,000	1 (3)	< $100,000	9 (30)
	$100,000 to $149,000	4 (13)	$100,000 to $150,000	5 (17)
	> $150,000	6 (20)	> $150,000	7 (23)
	Unreported	19 (63)	Unreported	9 (30)
	**Mean (SD)**		**Mean (SD)**	
**Birth Weight (kg) mean (SD)**	2.76 (0.27)		3.32 (0.46)	
**Weight-for-length Z-score mean (SD)**	-0.073 (0.96)		0.10 (0.93)	
**BMI Z-Score mean (SD)**	0.0091 (0.88)		0.46 (1.38)	
**BMI mean (SD)**	16.34 (1.56)		16.24 (2.27)	

### Quantitative results

The Parent Involvement Survey was self-administered to the parent immediately following completion of anthropometric measurements or within one week of their visit by electronic survey if they were unable to complete during the clinic visit. The survey was completed by thirty participants in each age group. Out of the 60 surveys that were self-administered, only five were completed electronically. 23 parents (76%) in the < 2 year age group and 28 parents (93%) in the 2–5 year age group rated their overall experience in being involved in their child’s anthropometric measurements as enjoyable or thoroughly enjoyable. Parents were very comfortable in assisting with their child’s anthropometric measurements (94% in < 2 years; 100% in 2–5 years) with 23 parents (77%) in the < 2 years and 28 parents (93%) in the 2–5 years, indicating they felt little or no stress from being involved. When parents were asked about their perspective of their child’s experience during their involvement in anthropometric measurements, of the < 2 year age group 19 parents (63%) believed their child felt little or no stress compared to 23 parents (77%), in the older age group. Only 40% of parents in both age groups reported previous experience assisting with clinical procedures, including anthropometric measurement and other procedures, in the healthcare setting. More than 80% of parents in both age groups expressed interest in being involved in their child’s anthropometric measurements in future clinic visits. All survey results are displayed in Table 1.

### Qualitative results

Open-ended responses of both age groups were analyzed separately, with common themes seen across both age groups. Thematic question analysis revealed five main themes as being key indicators of parent’s experience with participating in their child’s anthropometric measurements: [1] parent interest in child growth; [2] ease of measurement; [3] extended clinic visit; [4] minimizing child discomfort; and [4] interest in research participation.

### Theme 1: parent interest in child growth

This theme was defined by parent responses that included words or phrases surrounding the concept of learning about their child’s growth. Many parents expressed their interest in seeing their child’s growth progress and how their measurements had changed since a previous visit:

*“I enjoyed being involved and a part of obtaining the measurements of my child to see how the information was collected and changes over time.” (Parent)*

In addition, parents mentioned how they were interested in understanding child BMI, child growth charts, and how their child’s growth compares to the rest of the general population. Lastly, parents expressed their enjoyment in learning about the measurement collection process and the equipment used to take growth measurements.

### Theme 2: ease of measurement collection

This theme was defined by parent responses that included common words or phrases pointing towards the simplicity of the growth measurement collection. Parents mentioned that the process was quick, non-invasive, and easy to follow. Parents also expressed how accommodating and patient research staff were during the measurement collection.

*“The measurements were easy to take and took place during the doctor visits.” (Parent)*.

### Theme 3: extended clinic visit

This theme was defined by common words or phrases that attributed to the lengthening of the clinic visit itself. Common responses included the amount of paperwork to be completed, and how the parent’s participation in the study resulted in a lengthened time duration of the visit.

*“Sometimes the participation makes the doctor’s visits longer.” (Parent)*.

### Theme 4: minimizing child discomfort

This theme was defined by common words or phrases, including crying, frustration, lack of cooperation, fear and confusion, describing the child’s behaviour during the growth measurement collection. This theme was only seen in the parents of 0 – < 2 year age group.

*“Unfortunately, my son was getting frustrated with getting measured multiple times and was not cooperating well.” (Parent)*

### Theme 5: interest in research participation

This theme was defined by common words or phrases indicating parent’s enjoyment in being involved in a research study and their desire to contribute to research.

For example, one parent stated:

*“I enjoy participating in research studies with my child as I appreciate the chance to support scientific / health outcomes.” (Parent)*

Other parents expressed enjoyment with assisting the research assistant, as well as the desire to contribute to any future research.

## Discussion

Despite the importance of the parental role in the healthcare visit, no current literature is available on the parent’s experience of being involved in their child’s anthropometric measurements. This study showed that most parents enjoyed the experience of being involved in their child’s anthropometric measurements during their primary healthcare encounter, with higher enjoyment for children 2–5 years of age, compared to children < 2 years of age. Measurement of length for children who are < 2 years of age can be more stressful for both the parent and the child because of the different procedure followed when collecting their length measurement compared to the procedure for height measurement in children 2–5 years of age. Almost all parents reported that they were comfortable in assisting with their child’s anthropometric measurements. Finally, most parents said they experienced little to no stress during the measurements. Fewer parents of children younger than 2 years, compared to parents of children 2–5 years of age, felt that their presence provided a sense of comfort for their child.

A thematic analysis of open-ended questions on the parent’s overall experience revealed five major themes: [1] parent interest in child growth; [2] ease of measurement; [3] concerns about extending the clinic visit; [4] minimizing child discomfort; and [5] interest in research participation. Parents’ interest in child growth was similarly found in a previous Canadian-based study that investigated parents’ perception of their role in a pediatric intensive care unit within an affiliated university hospital [3]. In this study, it was reported that parents appreciated being informed of their child’s progress and treatment plan [3], similar to the parent reported interest in the measurement collection procedure and child growth progress over the years in the current study. It was anticipated that parents of both age groups would report that the measurement collection procedure was simple, quick, and non-invasive based on the protocol. It was also expected that parents would report that their visit was longer in duration due to the multiple sets of measurements collected. Lastly, parents involved expressed willingness and desire to contribute to future research with their children in the primary-care practice.

While most of the identified themes were expressed by parents of children from both age groups, the theme of ‘minimizing child discomfort’ was only identified in parent responses of the < 2 year age group. It is possible that the child discomfort was a result of procedural factors, and not a result of the parent involvement in the child’s anthropometric measurements, within the < 2 year age group. For example, many parents commented on the source of the child’s discomfort being frustration from multiple measurements. This effect would be mitigated outside of this research study when only two measurements would be performed. In addition, it was reported that the source of child discomfort was from the child’s clothes being removed during the weight measurement, which may have been related to the study procedural protocol. Lastly, when children are less than 2 years of age, the parent is not able to effectively provide an explanation of what type of procedure is being done, to help minimize the child’s discomfort through explanation. It is important to note that the source of parental stress during the healthcare encounter was not explored. There are various potential sources for parental stress during a healthcare encounter noted in the literature. One study on parental stress during immunization [11] found that children with a parent who rated themselves as reassuring, were less distressed during immunizations, compared to children with a parent that rated themselves as highly stressed prior to the procedures [11]. Other sources of parental stress may include pressure felt by the parent from working with a research assistant, measurement protocol complexity, or discomfort displayed by their child. Future research is needed to determine potential sources of parental stress during anthropometric measurements during a healthcare encounter using semi-structured interviews.

The present study has several strengths. Recruitment was conducted in a primary care setting, which resulted in a population of parents who often bring in their young children for anthropometric measurement collection. The survey design included both quantitative and qualitative components, which facilitated the identification of parent perception and feedback that may not have been captured by other research designs. The quantitative survey questions allowed for clear and scalable responses from the participants, whereas the qualitative open-ended questions allowed for more detailed, personalized responses regarding the parent’s overall experience. Data saturation was used to determine the desired sample size, which ensures that the data collected captured a wide array of diverse parent perspectives. Lastly, a thematic analysis provided a deeper understanding of the parent’s overall experience and allowed the development of overarching conceptual themes.

The study had several limitations. Researcher’s views and perspectives on participant responses are often implicit, which can result in bias when reviewing the survey questions. To help minimize this, the survey questions and responses were reviewed by a pair of additional researchers and parents to reduce the risk of biased interpretation. Participants may have answered the survey questions based on how they thought the researchers would like them to be answered, potentially resulting in false or inaccurate responses. Given the small sample size, the responses obtained in the survey may not be generalizable to other populations. In addition, although parents reported a high degree of enjoyment in being involved in their child’s measurement, their enjoyment (perceptions/approval) of not being involved (standard practice) was not evaluated. Lastly, it is important to note that the data were collected during the COVID – 19 pandemic, and participating research assistants were required to wear personal protective equipment (PPE) which may have caused increased fear or stress for the children.

## Conclusion

Parents report that their involvement in the collection of anthropometric measurement increased the security and comfort of their child during the procedure. Parent involvement may also benefit the parent themselves because of their reported interest in their child’s growth and measurements, and their desire to be involved in future primary healthcare visits. This study provides emerging evidence that supports the involvement of a parent with anthropometric measurements and provide feelings of inclusion for the parent. More research is needed in this area of parent involvement and understanding young children’s perspectives on their parent’s participation in the collection of anthropometric measurements may be the focus of further research in this area.

## Electronic supplementary material

Below is the link to the electronic supplementary material.

Supplementary Material 1

## Data Availability

All data generated or analyzed during this study are included in this published article.

## Abbreviations

PICU: Pediatric Intensive Care Unit
PPE: Personal Protective Equipment
TARGet Kids!: The Applied Research Group for Kids
WHO: World Health Organization
