# Supplementary material for: Parent involvement in child anthropometric measurement
Source: BMC Prim Care. 2023 Apr 5;24:89. doi: 10.1186/s12875-023-02028-2 (PMC10073622; doi:10.1186/s12875-023-02028-2)
Supplement: Supplementary file 1 — Supplementary Material 1 [file 12875_2023_2028_MOESM1_ESM.docx]

***TARGetKids! Collaborators** – Co-Leads: Catherine S. Birken, Jonathon L. Maguire; Advisory Committee: Ronald Cohn, Eddy Lau, Andreas Laupacis, Patricia C. Parkin, Michael Salter, Peter Szatmari, Shannon Weir; Science Review and Management Committees: Laura N. Anderson, Cornelia M. Borkhoff, Charles Keown-Stoneman, Christine Kowal, Dalah Mason; Site Investigators: Murtala Abdurrahman, Kelly Anderson, Gordon Arbess, Jillian Baker, Tony Barozzino, Sylvie Bergeron, Dimple Bhagat, Gary Bloch, Joey Bonifacio, Ashna Bowry, Caroline Calpin, Douglas Campbell, Sohail Cheema, Elaine Cheng, Brian Chisamore, Evelyn Constantin, Karoon Danayan, Paul Das, Mary Beth Derocher, Anh Do, Kathleen Doukas, Anne Egger, Allison Farber, Amy Freedman, Sloane Freeman, Sharon Gazeley, Charlie Guiang, Dan Ha, Curtis Handford, Laura Hanson, Leah Harrington, Sheila Jacobson, Lukasz Jagiello, Gwen Jansz, Paul Kadar, Florence Kim, Tara Kiran, Holly Knowles, Bruce Kwok, Sheila Lakhoo, Margarita Lam-Antoniades, Eddy Lau, Denis Leduc, Fok-Han Leung, Alan Li, Patricia Li, Jessica Malach, Roy Male, Vashti Mascoll, Aleks Meret, Elise Mok, Rosemary Moodie, Maya Nader, Katherine Nash, Sharon Naymark, James Owen, Michael Peer, Kifi Pena, Marty Perlmutar, Navindra Persaud, Andrew Pinto, Michelle Porepa, Vikky Qi, Nasreen Ramji, Noor Ramji, Danyaal Raza, Alana Rosenthal, Katherine Rouleau, Caroline Ruderman, Janet Saunderson, Vanna Schiralli, Michael Sgro, Hafiz Shuja, Susan Shepherd, Barbara Smiltnieks, Cinntha Srikanthan, Carolyn Taylor, Stephen Treherne, Suzanne Turner, Fatima Uddin, Meta van den Heuvel, Joanne Vaughan, Thea Weisdorf, Sheila Wijayasinghe, Peter Wong, John Yaremko, Ethel Ying, Elizabeth Young, Michael Zajdman; Research Team: Farnaz Bazeghi, Marivic Bustos, Charmaine Camacho, Dharma Dalwadi, Christine Koroshegyi, Tarandeep Malhi, Sharon Thadani, Julia Thompson, Laurie Thompson; Project Team: Mary Aglipay, Imaan Bayoumi, Sarah Carsley, Katherine Cost, Anne Fuller, Laura Kinlin, Jessica Omand, Shelley Vanderhout, Leigh Vanderloo; Applied Health Research Centre: Christopher Allen, Bryan Boodhoo, Olivia Chan, David W.H. Dai, Judith Hall, Peter Juni, Gerald Lebovic, Karen Pope, Kevin Thorpe; Mount Sinai Services Laboratory: Rita Kandel, Michelle Rodrigues.
